# Supplementary material for: Prolonged cooling and degassing of Apollo 17 volcanic glasses on the lunar surface
Source: Nat Commun. 2026 Feb 3;17:2291. doi: 10.1038/s41467-026-69087-8 (PMC12976169; doi:10.1038/s41467-026-69087-8)
Supplement: Supplementary file 1 — Supplementary Information [file 41467_2026_69087_MOESM1_ESM.pdf]

Supplementary Materials for:

Prolonged cooling and degassing of Apollo 17 volcanic glasses on the lunar surface

Peng Ni[1], Yan Zhan[2]

[1]*Department of Earth, Planetary, and Space Sciences, University of California, Los Angeles, CA 90095, USA*

[2]*Department of Earth and Environmental Sciences, The Chinese University of Hong Kong, Shatin, N.T., Hong Kong*

This PDF file includes:

Supplementary discussions
Supplementary references
Supplementary Figures S1 to S14
Supplementary Table S1 and S2

Supplementary discussions

1. Modeling H_2O, F, Cl, and S concentrations in the orange glass beads assuming volatile degassing occurred solely during free flight

We conducted a series of sensitivity tests to calculate the loss of volatiles (H_2O, Cl, F, and S) from glass beads during free flight. In these tests, the cooling timescale (τ_c) ranged from 10 to 100,000 seconds, and the dimensionless parameter L_i (the ratio between diffusion and evaporation timescales) ranged from 10^{-3} to 10^3. We also varied the radius of the glass bead models from 50 to 150 μm to match the observations.

The concentrations of volatiles in a spherical glass bead were calculated using the equations provided in the Methods section (Eq. M7–M9). To represent the bulk volatile concentration of the glass beads, the volumetric average concentration of volatiles within 50% of the bead radius was calculated.

The results show a clear trend: longer cooling timescales lead to greater volatile losses. For models where diffusion timescales are shorter than evaporation timescales (L_i<1), volatile loss increases as L_i becomes larger. In contrast, for models where diffusion timescales are significantly longer than evaporation timescales, the parameter L_i has minimal influence, indicating that diffusion within the glass bead becomes the rate-limiting step (Fig. S2).

To explain the observed glass bead data (histograms next to the color bars in Fig. S2), the minimum cooling timescale required for different bead sizes is approximately 10^2~10^3 seconds for high-diffusivity elements like H_2O, and 10^3~10^4 seconds for low-diffusivity elements like S. Thus, to account for the volatile loss of all four elements with a consistent thermal history, the minimum cooling timescale is about 10^3 seconds for small glass beads (50 μm) and about 10^4 seconds for large glass beads (150 μm).

In an extreme scenario, we assumed that sulfur concentrations in the glass beads were directly inherited from the magma during upwelling, with no diffusive sulfur loss occurring during free flight. We calculated the relative loss of other elements compared to sulfur (C_i^{rel}, i = H_2O, Cl, or F) using $C_i^{rel} = (C_i/C_S)/(C_i^{Stage1}/C_S^{Stage1})$, in which the ratios between volatile elements and sulfur ($C_i^{Stage1}/C_S^{Stage1}$) immediately after fragmentation (before taking off) were determined from the melt inclusion trends (Fig. 3).

The results (Fig. S3) are consistent with those in the previous scenario (Fig. S4). To explain the data, the required cooling timescale during free flight is at least 10^3 ~ 10^4 seconds for different sizes of glass beads.

2. Validation of the 1D semi-analytical model for melt embayment degassing

In order to validate the semi-analytical approach we adopted for modeling volatile degassing in the melt embayment, numerical simulations using COMSOL Multiphysics were also conducted for comparison. We modeled degassing of a hypothetic element with initial concentration of C_0, diffusivity (D) of $4.68 \times 10^{-11}\ m^2/s$, and surface evaporation rate (v_i) of $10^{-7.2}\ m/s$ at a constant temperature and tracked the profile of C/C_0 as a function of time.

One-dimensional simulation in COMSOL was conducted using the governing diffusion equation given in Eq. 5 and boundary conditions defined by Eqs. 9 and 10. A 2D finite

element model was designed to capture the actual geometry of the melt embayment (Fig. S4a). By assuming the embayment to be rotationally symmetric around its longitudinal axis, its three-dimensional geometry gets reduced to a two-dimensional cross-section (Fig. S4a). The longitudinal axis represents the primary direction of diffusion, while the radial direction accounts for lateral diffusion. This simplification maintains the essential geometry while significantly reducing computational costs.

The governing equation for diffusion in 2D axial symmetric system can be described by:

$$\frac{\partial C}{\partial t} = D\left(\frac{\partial^2 C}{\partial z^2} + \frac{1}{r}\frac{\partial}{\partial r}\left(r\frac{\partial C}{\partial r}\right)\right). \quad \text{(S1)}$$

Here C is the concentration in the embayment as a function of radial (r) and longitudinal (z) positions through time (t) and D is the diffusivity of the volatile component.

The boundary conditions are defined as:

$$\frac{\partial C}{\partial n} = -\upsilon C \quad \text{(S2)}$$

for the open side of the embayment (Fig. S4a) and

$$\frac{\partial C}{\partial n} = 0 \quad \text{(S3)}$$

for the surface in contact with olivine.

In Eqs. S2 and S3, υ is the evaporation rate of the component and $\frac{\partial C}{\partial n}$ is the concentration gradient normal to the embayment surface. Axial symmetry was enforced along the longitudinal axis and the embayment was assumed to be initially homogeneous in composition. For solving Eqs. S1–S3, the domain was discretized into a fine triangular mesh (Fig. S4b) and adaptive time-stepping was used to ensure numerical stability and accuracy, particularly during rapid changes in diffusivity or concentration.

Results of the 1D semi-analytical model are plotted and compared with those of the 1D or 2D axisymmetric finite element model in Fig. S4c for comparison. Consistent results were obtained between the 1D semi-analytical approach and the 1D finite element model using COMSOL. Modeled concentrations along the longitudinal axis of the embayment in the 2D axisymmetric approach were mostly within 15% of the 1D results. While the 2D axisymmetric numerical model provides additional precision, the small differences justify the 1D simplification. A simplified 1D approach effectively captures the diffusion process, which is dominated by the longitudinal direction, while offering incomparable computational efficiency.

3. One-dimensional thermal evolution model for Apollo 17 pyroclastic deposit

To investigate the thermal evolution of the Apollo 17 pyroclastic deposit, particularly for sample 74220, we developed a one-dimensional (1D) transient heat transfer model. The model simulates the cooling of a pyroclastic deposit on the lunar surface, accounting for heat conduction within the deposit, radiative heat loss to space, and periodic solar heating due to the lunar diurnal cycle. The model domain is a 1D interval representing a 2-meter-thick pyroclastic deposit. This thickness was chosen to encompass the estimated burial depth of sample 74220 (~30 cm) while allowing sufficient depth to capture subsurface thermal gradients and the influence of a stable subsurface temperature.

The heat transfer physics was modeled using the COMSOL Multiphysics, solving the 1D transient heat conduction equation:

$$\rho_r C_p \frac{\partial T}{\partial t} = \frac{\partial}{\partial t}\left(k_r(T)\frac{\partial T}{\partial z}\right), \tag{S3}$$

where ρ_r is the density of the pyroclastic deposit (1500 kg/m^3), C_p is the specific heat capacity (600 J/kg/K), and $k_r(T)$ is the thermal conductivity. In the models with constant k_r, the k_r is set to be 1 mW/m/K, consistent with the values for lunar regolith (0.5–2 mW/m/K) and simulants (1–2 mW/m/K at ~300 K)[1]. Additionally, a temperature-dependent thermal conductivity was also employed to examine its effect on the cooling history. The temperature-dependent thermal conductivity function was defined as:

$$k_r(T) = 5 \times 10^{-4} \times \left(\max\frac{(T,300)}{300}\right)^3 W/m/K. \tag{S4}$$

This function accounts for the increase in thermal conductivity with temperature due to radiative contributions within the porous regolith, capped at a minimum of 300 K to avoid unphysical behavior at low temperatures.

Boundary conditions include a thermal insulation at the bottom (2 m) for simplification, and a surface-to-ambient radiation at z = 0 m, with an emissivity of 0.95 and an ambient temperature of 2.7 K, following the Stefan-Boltzmann law:

$$q_{rad} = \epsilon\sigma\left(T^4 - T_{space}^4\right), \tag{S5}$$

where σ is the Stefan-Boltzmann Constant (5.67 × 10^{-8} W/m^2/K^4). The emissivity (ϵ) is 0.95, reflecting the high emissivity of lunar regolith surfaces, which efficiently radiate heat to space. The space temperature (T_{space}) is 2.7 K. In some tests, we also considered the solar heat flux at the surface (z = 0 m):

$$q_{solar}(t) = \frac{S_{solar}(1-Albedo)}{2}\cos\left(\frac{2\pi t}{P_{moon}}\right), \tag{S6}$$

where the solar constant (S_{solar}) is 1361 W/m^2, representing the average solar flux at the Moon’s orbit. An albedo value of 0.12 was adopted for the model, which is typical for the lunar surface, accounting for the fraction of reflected solar radiation. The diurnal cycle for solar heating was defined using the lunar day period (P_{moon}) of 29.5 Earth days.

The geometry was discretized using a mesh with 100 elements, refined near the surface (element ratio of 50) to resolve steep temperature gradients caused by solar heating and radiative cooling. The simulation was run over a total time of 10 years to evaluate long-term cooling trends, with time steps scaled to capture both diurnal variations and long-term evolution.

We tested four scenarios by considering constant or temperature-dependent thermal conductivities, with or without solar heat input (Fig. S6). For the models that included solar heat input, the surface temperature exhibits a long-term diurnal oscillation ranging from 100 K to 350 K, which is consistent with observations of lunar surface temperatures, demonstrating the robustness of the pyroclastic deposit cooling model. In the more realistic scenario (temperature-dependent k_r and solar heating), temperatures remain relatively high even after several years. At a depth of 30 cm, the temperature is approximately 600 K after three years of cooling and ~475 K after a decade. These tests primarily serve to demonstrate

that years of cooling time is not an unreasonable assumption for fire fountain eruptions that accumulated hot pyroclastic deposits in short time periods.

4. Forward modeling for sulfur "ingassing" in Apollo 17 orange glass beads

To simulate sulfur ingassing into the Apollo 17 orange glass beads[2], we applied an analytical solution for one-dimensional spherical diffusion with a constant surface concentration (Dirichlet boundary condition). The forward model computes the temporal and spatial evolution of sulfur concentration within a spherical melt droplet during post-eruptive cooling and exposure to the surrounding gas phase.

The sulfur concentration profiles, $C_i(r, 0)$, for each glass bead at the end of three-stage degassing were derived from the posterior ensemble mean of the three-stage glass bead degassing model with data assimilation (see Methods). Subsequently, a subset of ensemble models with central sulfur concentrations that match the measured ingassing profiles in orange glass beads was selected and averaged to serve as the initial sulfur concentration profile for ingassing. (Figure S13; Su et al.[2]).

During the ingassing stage, the sulfur concentration at the bead surface (C_b) is assumed to remain constant, representing equilibrium with a layer of condensed sulfide on the surface. The values of C_b for the glass beads with different radii are about 500 ppm determined by the highest concentrations along the profiles (Figure S13). The analytical solution for this constant boundary condition is given by:

$$C(r,t) = C_b + \frac{2}{r}\sum_{n=1}^{N}\left[\frac{aC_b\cos(n\pi)}{n\pi} + \frac{2}{a}\int_0^a r'\, C_i(r')\sin\left(\frac{n\pi r'}{a}\right)dr'\right]\sin\left(\frac{n\pi r}{a}\right)\exp(-Dn^2\pi^2 t/a^2) \qquad \text{(S7)}$$

where *a* is the radius of the spherical bead. The integral term accounts for the non-uniform initial concentration profile obtained from the data-assimilated three-stage model results. This formulation allows the sulfur concentration in the bead to be evaluated as a function of both radial position (*r*) and time (*t*).

The resulting radial sulfur concentration profiles, $C(r, t_x)$, were calculated at the glass-transition temperature of 938 K and compared directly with the measurements[2].

5. Forward modeling for the degassing of Apollo 15 green glass beads

Due to the lack of olivine-hosted melt inclusions in the Apollo 15 pyroclastic deposits, the degassing history for the green glass beads is poorly constrained. To assess the potential impact of prolonged lunar surface cooling on the volatile loss in green glass beads, we conducted forward modeling assuming a cooling history similar to that of the orange glass beads.

The forward model calculates volatile concentrations (H_2O, F, Cl, S) across three stages, using parameters derived from the EnKF test for the orange glass beads (Figure S12). Similar

to the orange glass beads, Stage 1 (decompression) models initial volatile exsolution during magma ascent. Stage2 (free-flight) simulates hyperbolic cooling from a higher initial temperature using its liquidus (T_0 = 1723 K)[3] to a lower glass transition temperature (T_g = 883 K)[4], incorporating constant evaporation governed by a diffusion-evaporation ratio (L parameter), with a fixed free flight time scale. Stage 3 (cooling on lunar surface) assumes continuous evaporation at the constant T_g and the same L value as Stage 2 for a fixed duration.

The results for a 150-µm-radius green glass bead, modeled using the above three-stage degassing process, reveal distinct patterns in volatile concentration distributions (Figure S12). For H_2O, the ensemble distribution peak around 10^{-4}, indicating complete degassing during Stage 3. While for F and Cl, the volatile losses are moderate. Sulfur is expected to have the highest residual concentration after degassing. Such extreme degassing is inconsistent with the observations for green glass beads, indicating that the green glass beads likely cooled down faster than orange glass beads on the lunar surface. This was either due to a shallower burial depth, or slower burial rate that allowed for faster cooling of the pyroclastic deposit at the Apollo 15 sampling site.

Supplementary references

1. Sakatani, N., Ogawa, K., Arakawa, M. & Tanaka, S. Thermal conductivity of lunar regolith simulant JSC-1A under vacuum. *Icarus* **309**, 13–24 (2018).
2. Su, X., Zhang, Y. & Liu, Y. Sulfur outgassing and in-gassing in lunar orange glass beads and implications for 33S "Anomaly" in the Moon. *Geochimica et Cosmochimica Acta* **397**, 164–175 (2025).
3. Barr, J. A. & Grove, T. L. Experimental petrology of the Apollo 15 group A green glasses: Melting primordial lunar mantle and magma ocean cumulate assimilation. *Geochimica et Cosmochimica Acta* **106**, 216–230 (2013).
4. Arndt, J. & von Engelhardt, W. Formation of Apollo 17 orange and black glass beads. *J. Geophys. Res.* **92**, E372–E376 (1987).
5. Freda, C., Baker, D. R. & Scarlato, P. Sulfur diffusion in basaltic melts. *Geochimica et Cosmochimica Acta* **69**, 5061–5069 (2005).
6. Alletti, M., Baker, D. R. & Freda, C. Halogen diffusion in a basaltic melt. *Geochimica et Cosmochimica Acta* **71**, 3570–3580 (2007).
7. Newcombe, M. E. *et al.* Effects of pH2O, pH2 and fO2 on the diffusion of H-bearing species in lunar basaltic liquid and an iron-free basaltic analog at 1 atm. *Geochimica et Cosmochimica Acta* **259**, 316–343 (2019).
8. Zhang, L. *et al.* Reassessment of pre-eruptive water content of lunar volcanic glass based on new data of water diffusivity. *Earth and Planetary Science Letters* **522**, 40–47 (2019).

Supplementary figures

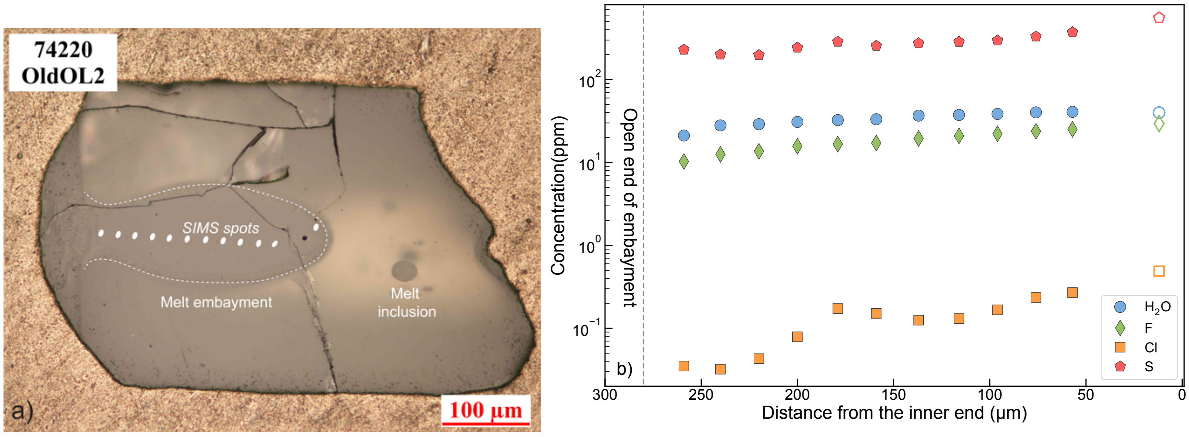

Figure S1. Microscopic image (a) and measured volatile concentration profiles (b) in the melt embayment of 74220 OldOl2. Locations of the ion beam for spot analyses are marked with circles on the microscopic image. The melt embayment is outlined with the white dashed curve. The analytical point closest to the inner end of the melt embayment (open symbols in b) was too close to the exsolved bubble and therefore excluded for modeling. Concentrations plotted in b are not corrected for the effect of post-entrapment crystallization.

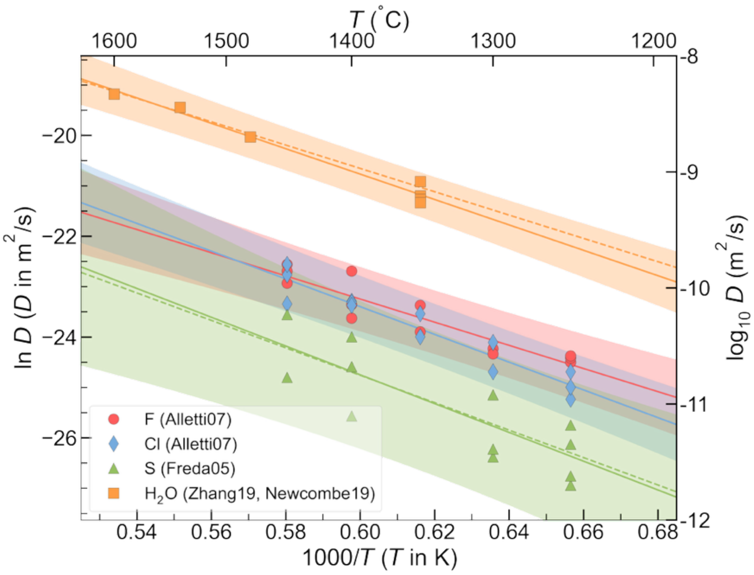

Figure S2. Summary of experimental data for H_2O, F, Cl, and S diffusivities used for modeling in this study[5–8]**.** Solid curves represent the best fit curve, and the colored regions represent 95% confidence interval for the fit. Constants obtained from the regression are provided in Table S1. Expressions for H_2O diffusivity from Zhang et al.[8] and S diffusivity from Freda et al.[5] are plotted in dashed curves for comparison.

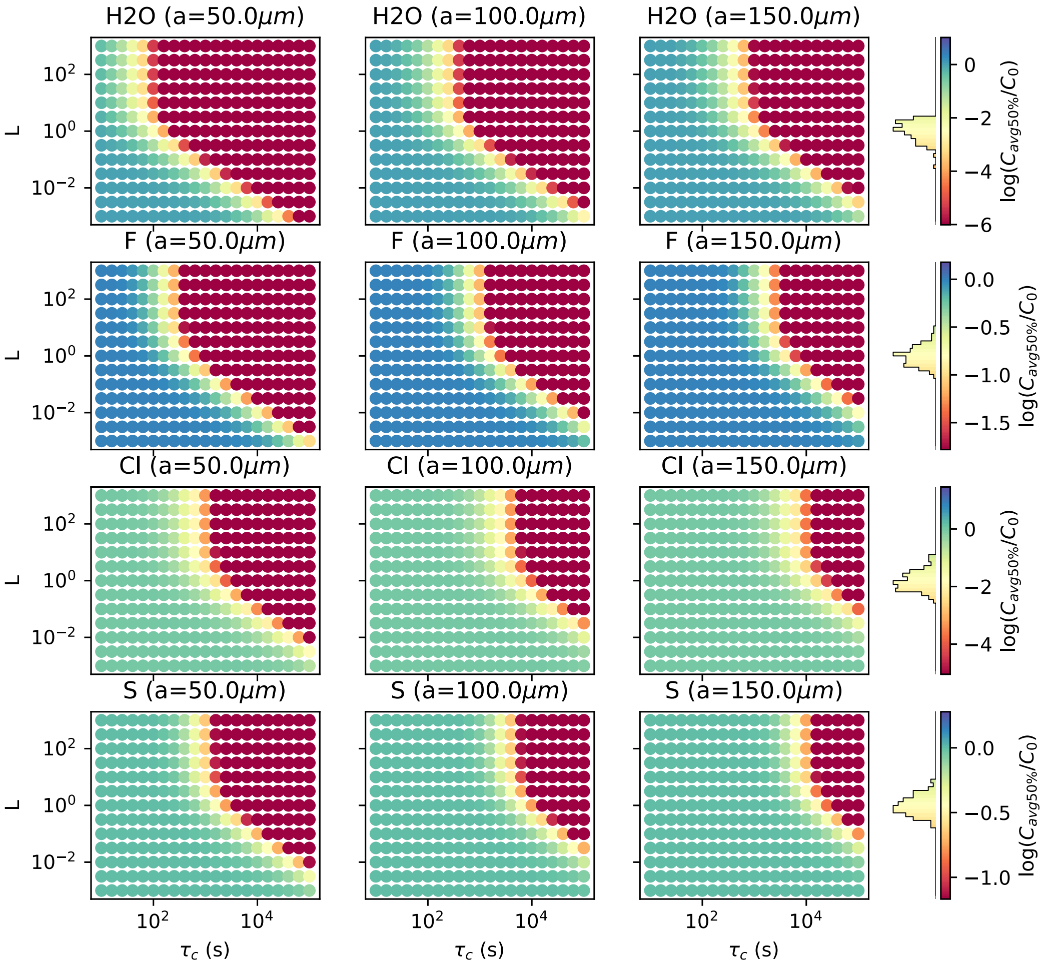

Figure S3. Comparison of the absolute volatile loss between the model and the data. The colored dots represent the results of sensitivity tests for glass bead concentration modeling. The histograms next to the color bar show the distribution of volatile concentrations in the glass beads. The color maps show the volumetric average concentration of the volatiles within 50% of the radius of the glass beads, modeled using different bead radii (a), characteristic cooling time scales (τ_c), and the ratio between diffusion and evaporation time scales (L_i).

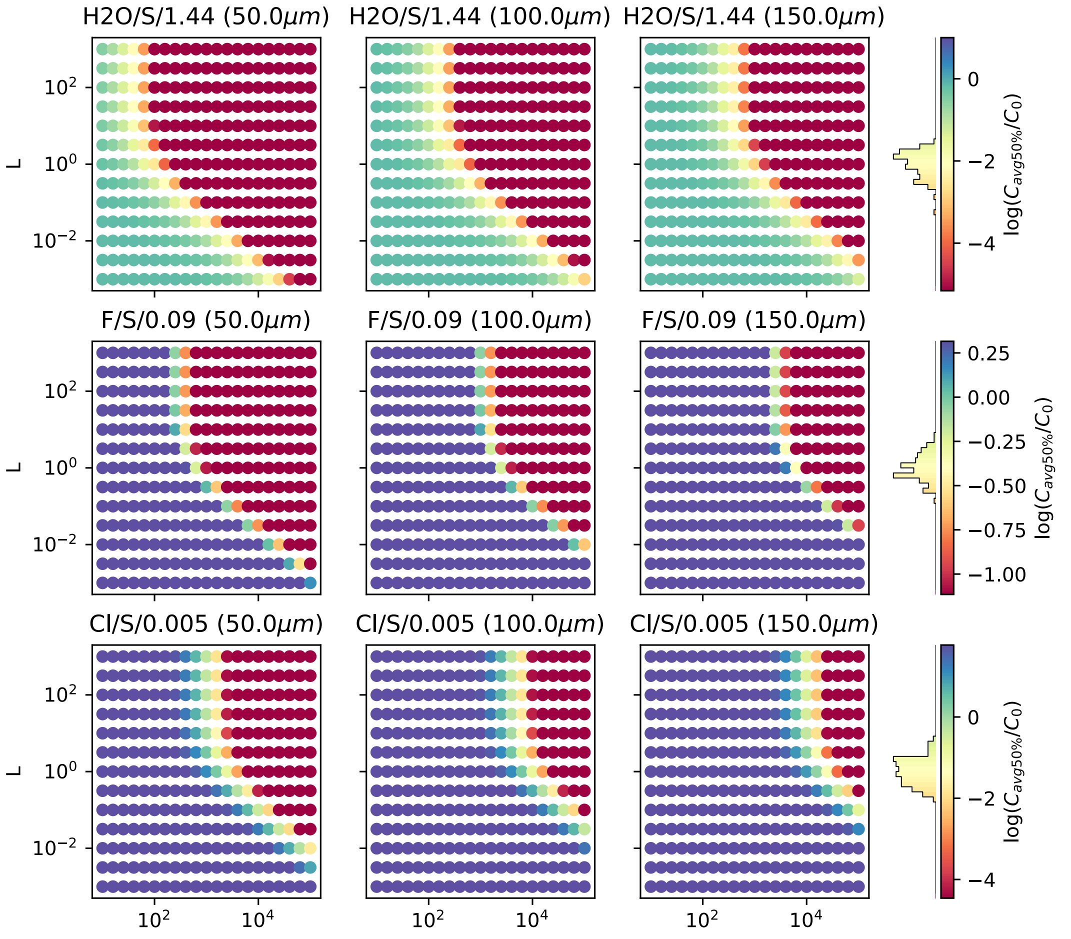

Figure S4. Comparison of the relative volatile loss between the model and the data. The colored dots represent the results of sensitivity tests for glass bead concentration modeling. The histograms next to the color bar show the distribution of volatile concentrations in the glass beads. The legend and color bar are similar to those in Figure S2.

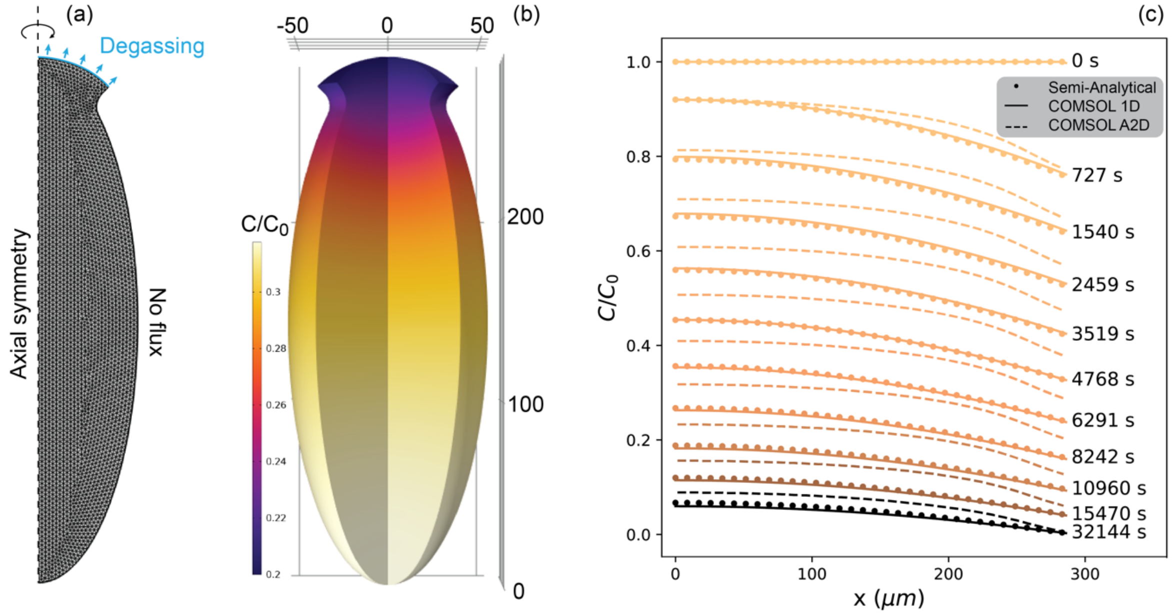

Figure S5. Validation of the semi-analytical model with finite element numerical modeling. (a) Setup of the two-dimensional (2D) axisymmetric model for the melt embayment. (b) Normalized concentration distribution in the embayment after 8242 seconds of diffusive loss for the 2D axisymmetric model. (c) Comparison of modeled profiles from the semi-analytical model with numerical results for 1D and 2D axisymmetric setups using COMSOL Multiphysics (see Supplementary discussion 2).

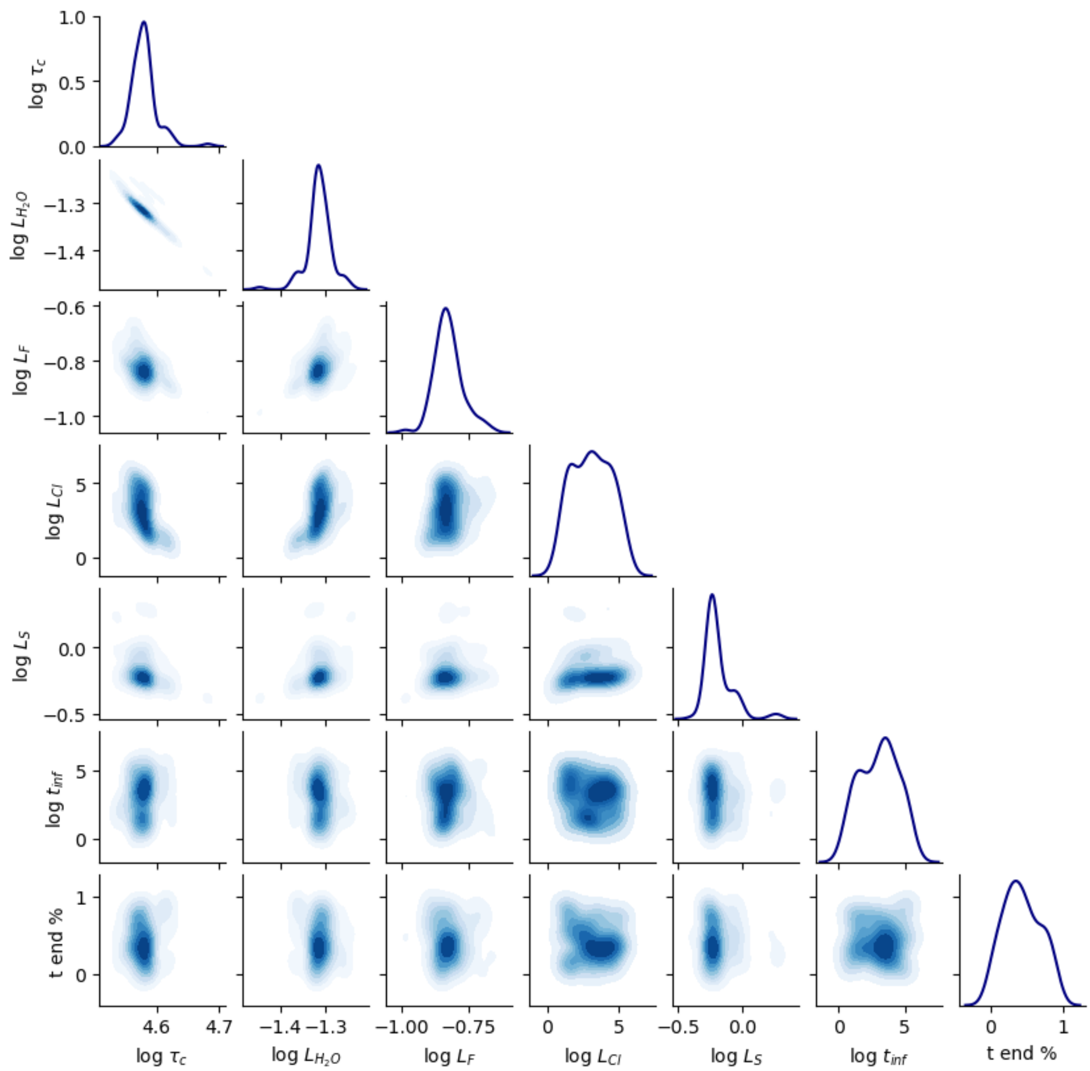

Figure S6. The joint and marginal distributions of the model parameters from the two-stage degassing model for melt embayment degassing using Ensemble Kalman Filter (EnKF) assimilation. The colored contours show the kernel density distribution of different parameter pairs, while the diagonal subplots display the histograms of individual parameters. Here t_{end} is defined as a percentage of t_{∞}. Details of the model are provided in Methods. This two-stage model assumes that degassing loss occurred only during magma upwelling (Stage 1) and post-eruption free flight (Stage2). Degassing loss on the lunar surface (Stage 3) is not considered. However, such a two-stage model would require an unrealistically long cooling time scale (τ_c) for Stage 2.

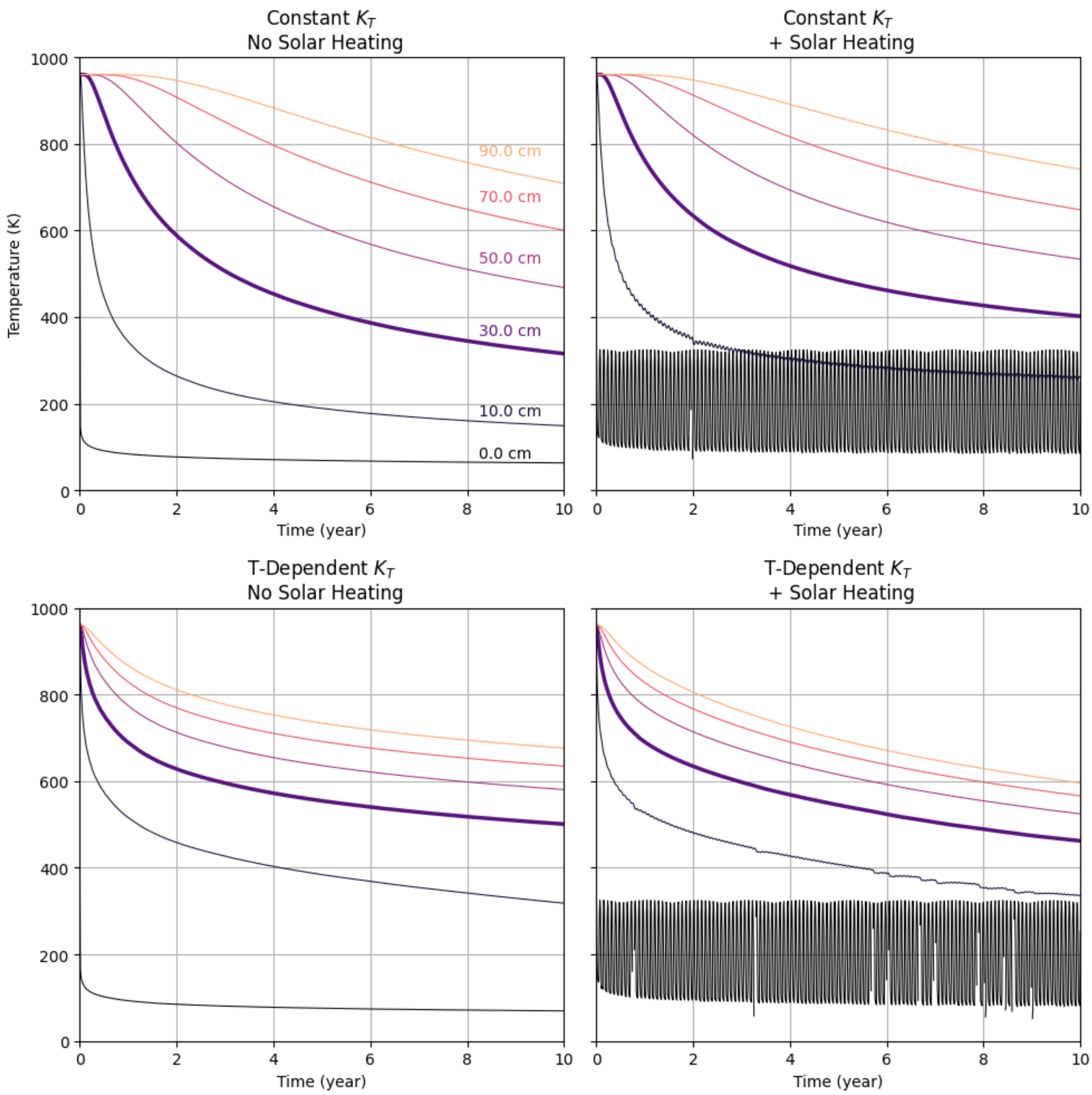

Figure S7. The cooling history of the pyroclastic deposits at depths ranging from the surface to 90 cm. The dashed, solid, and dotted lines represent models using constant thermal conductivity (1 mW m^{-1} K^{-1}), constant properties with solar heating, and temperature-dependent thermal conductivity, respectively. The burial depth of lunar sample 74220 is estimated to be ~30 cm based on the Apollo 17 Preliminary Science Report.

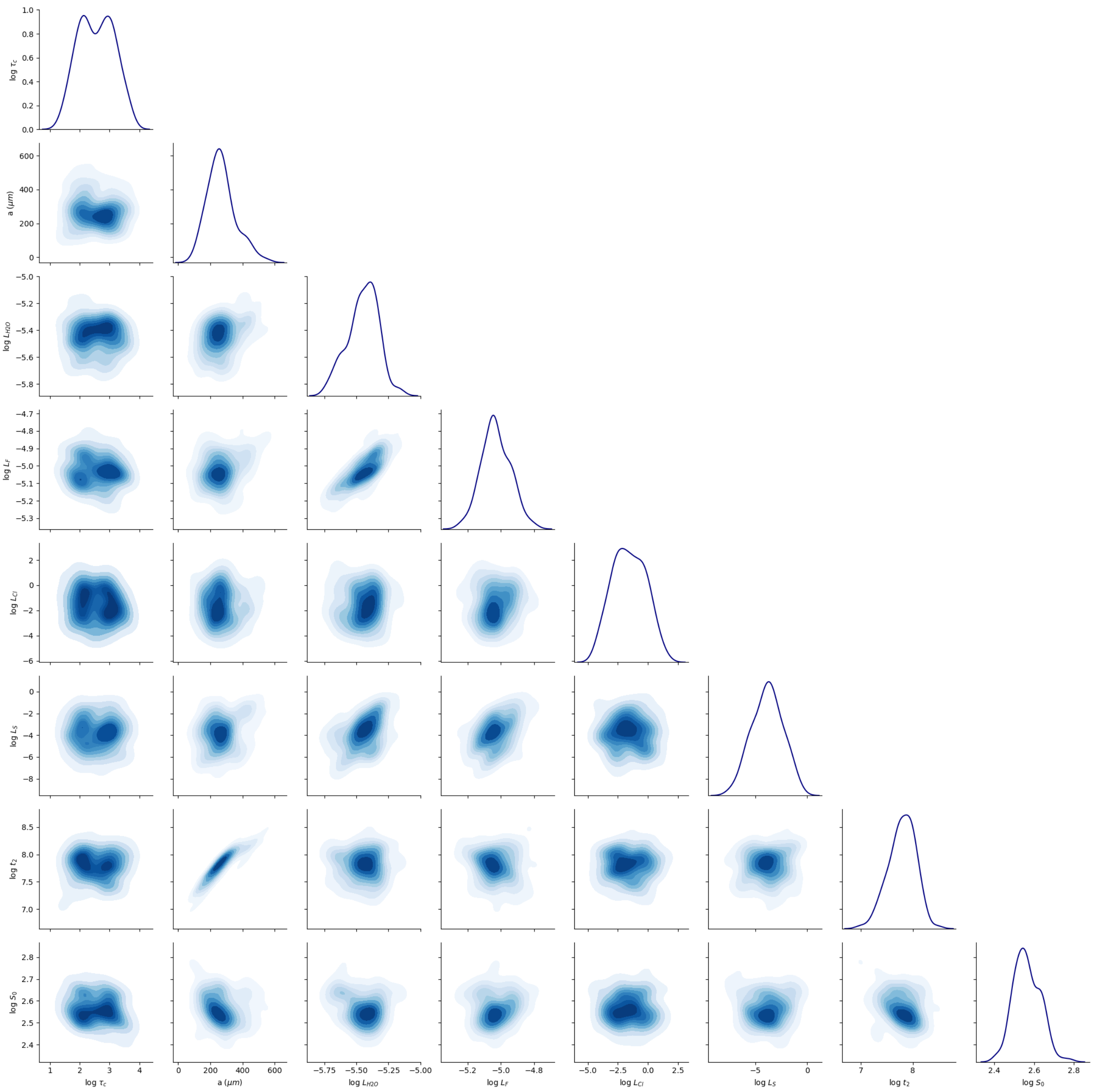

Figure S8. The joint and marginal distributions of the model parameters from the three-stage degassing model for orange glass beads using Ensemble Kalman Filter (EnKF) assimilation. The 5x5 grid includes 2D histograms (blue contours) showing pairwise correlations and 1D marginal histograms (blue curves) along the diagonal, illustrating the distribution of each variable. The contour density reflects the probability density of ensemble members, highlighting the refined relationships and uncertainties in volatile and size distributions post-assimilation.

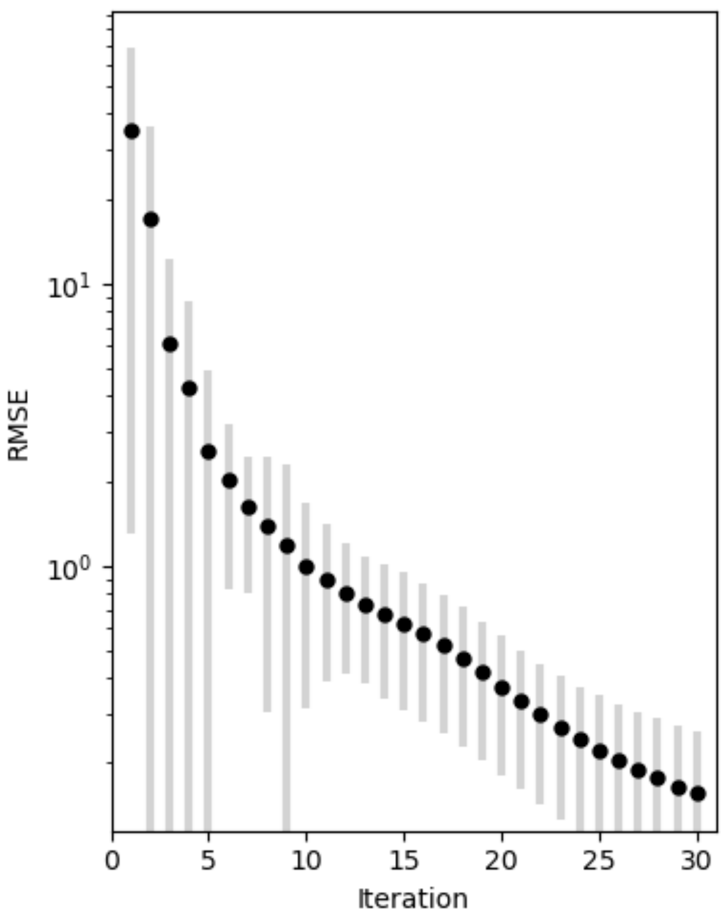

Figure S9. The evolution of the root mean square error (RMSE) of volatile concentrations of the glass beads between the model and data (Figure 3) in the data assimilation.

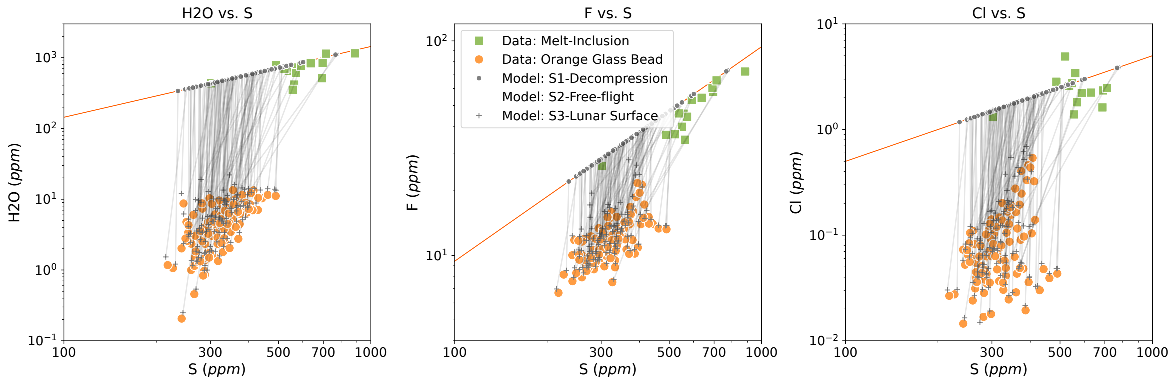

Figure S10. The evolution of volatile concentrations (H_2O, F, Cl) versus sulfur (S) in lunar glass beads, modeled using the three-stage EnKF assimilation. Panels show volatile concentration data from orange glass beads (orange circles) and melt inclusions (green squares), and model stages: Stage 1 (Decompression, grey circle), Stage 2 (Free flight, overlap with Stage 1, not shown), Stage 3 (Deposition, black plus). Gray curves indicate the trajectories for volatile loss in the three-stage model.

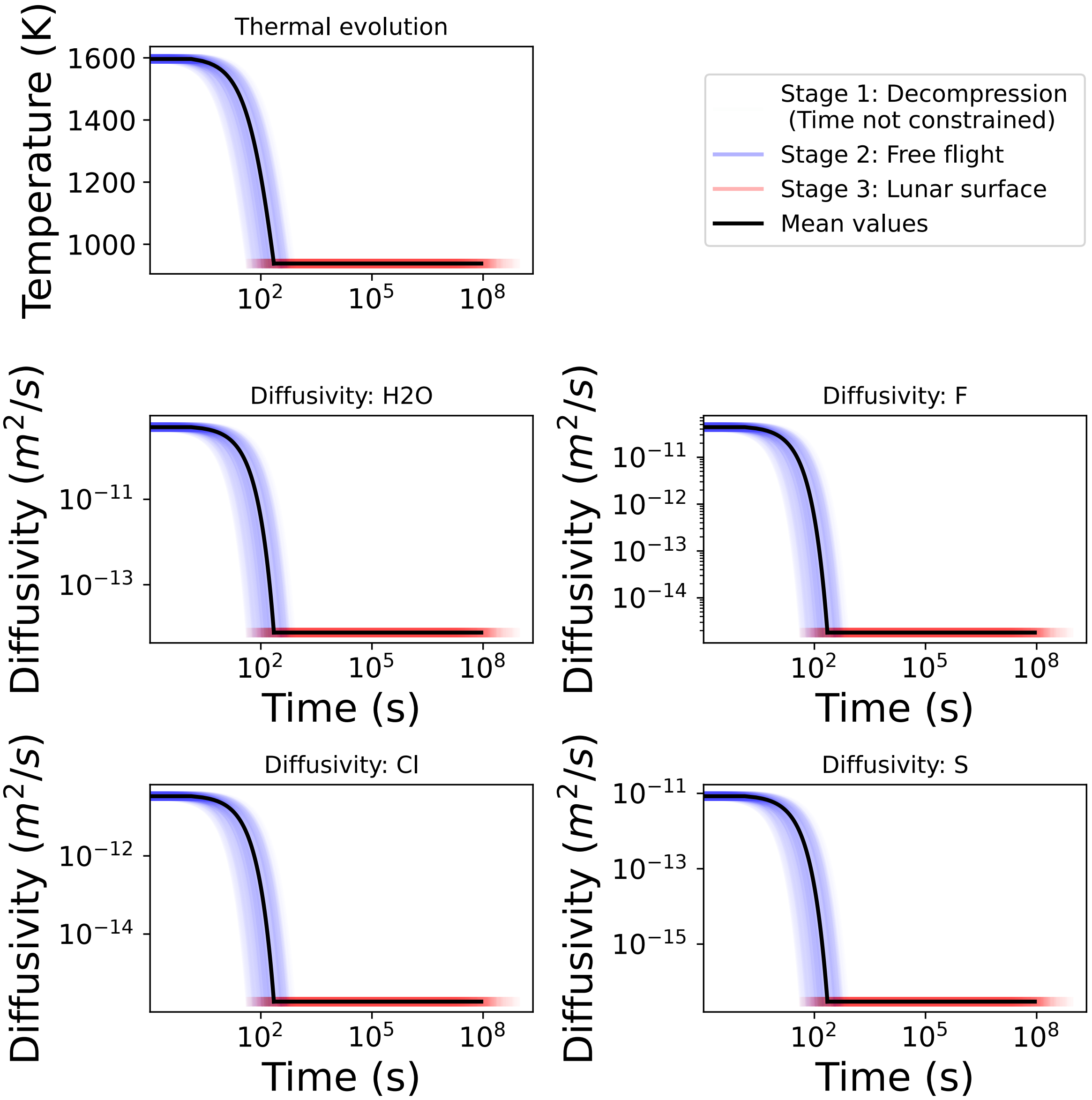

Figure S11. Thermal history and time-dependent diffusivities obtained from data assimilation of the three-stage glass bead degassing model. Parameters for the thermal history are from Fig. S8. Equations for calculating diffusivities as a function of temperature are reported in Table S1.

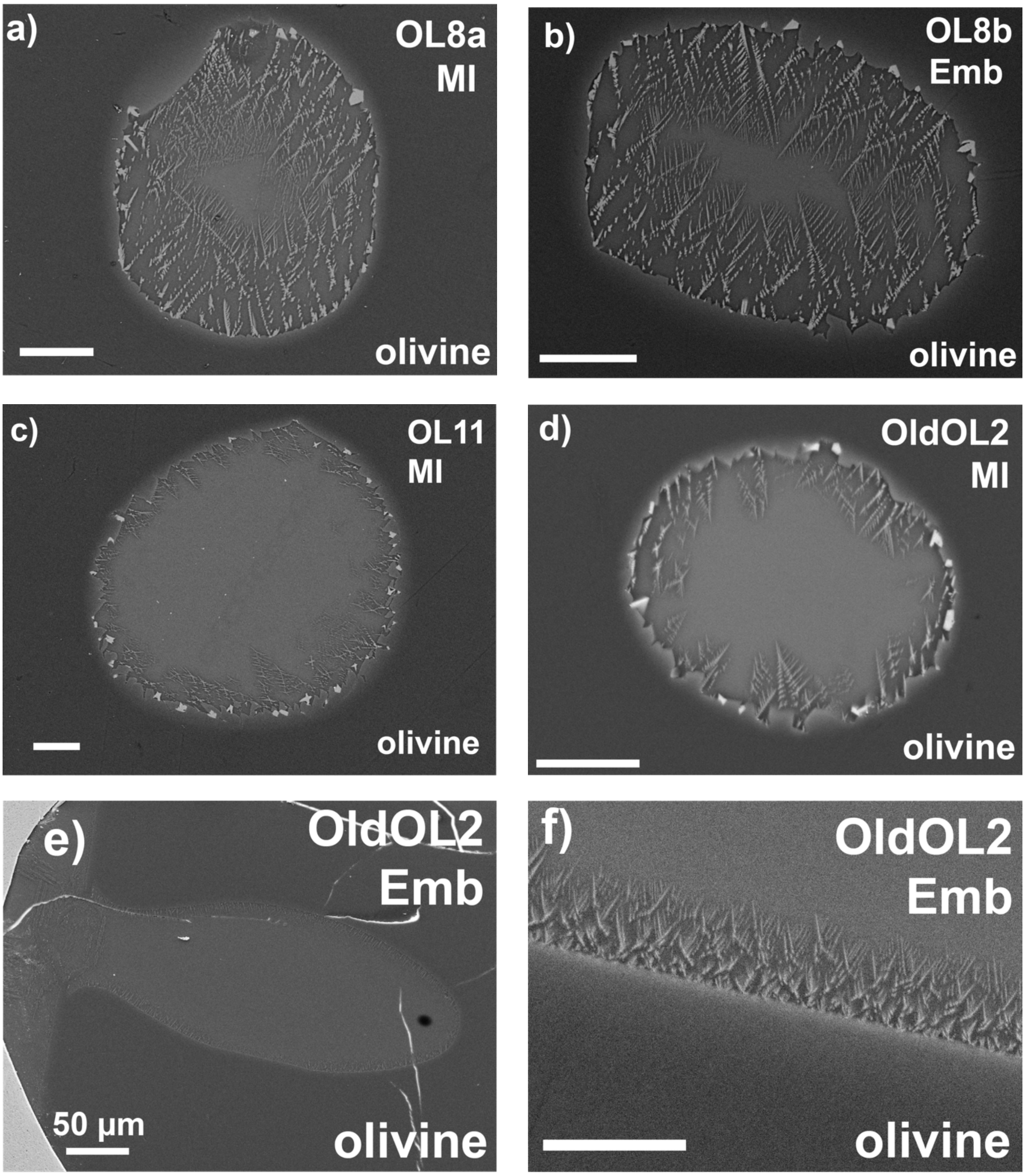

Figure S12. Back-scattered electron images of olivine-hosted melt inclusions (a, c, d) and melt embayments (b, e, f) from lunar sample 74220. Virtually all melt inclusions and embayments exhibit dendritic olivine growth from the melt–olivine interface, indicating an additional stage of olivine crystallization beyond concentric post-entrapment olivine growth. Some of the inclusions also contain bright ilmenite crystals.

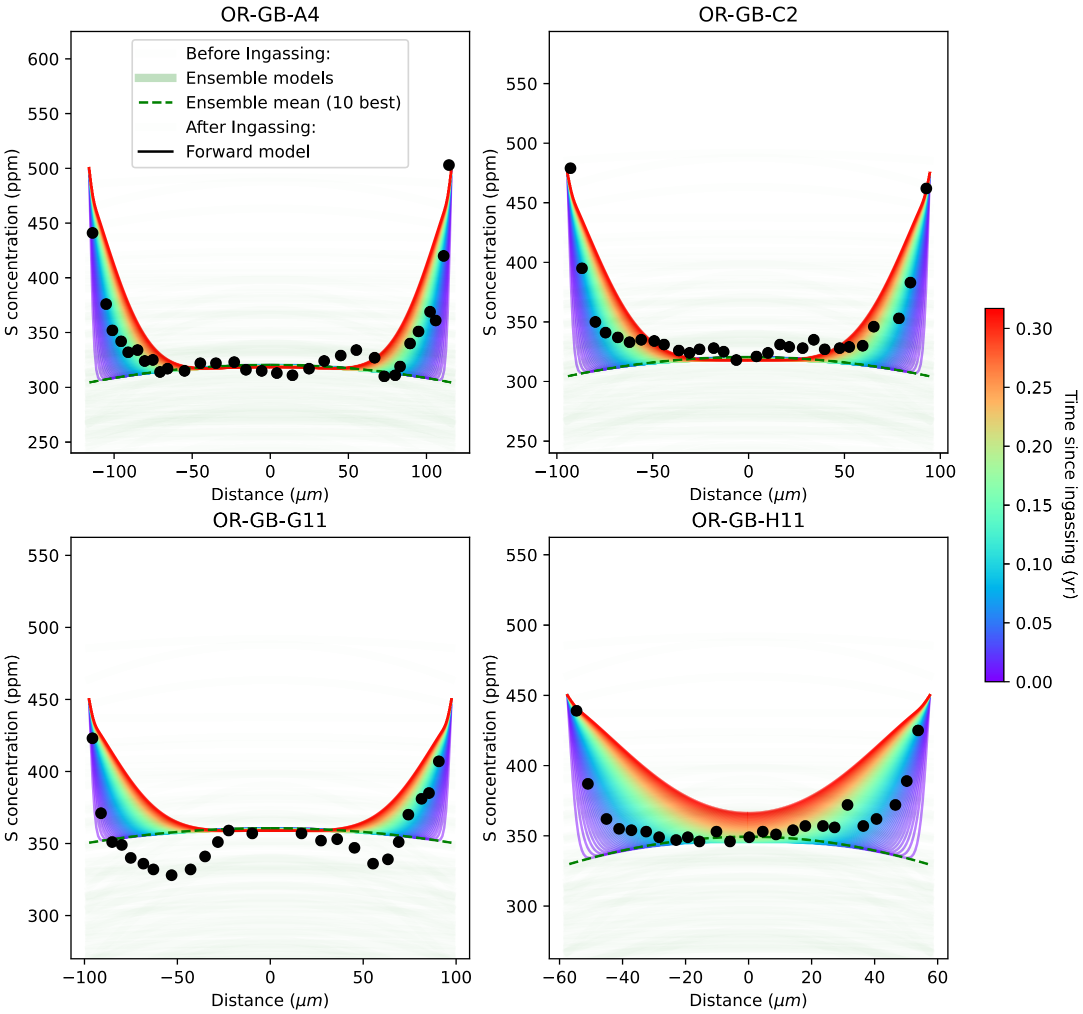

Figure S13. Forward modeling to reproduce the U-shaped and W-shaped S "ingassing" profiles found in orange glass beads. The sulfur concentration profiles are from Su et al.[2]. The results show that these profiles can also be produced by solid-state diffusion at 938 K for a month or shorter. Details of the model are provided in Supplementary discussion 4.

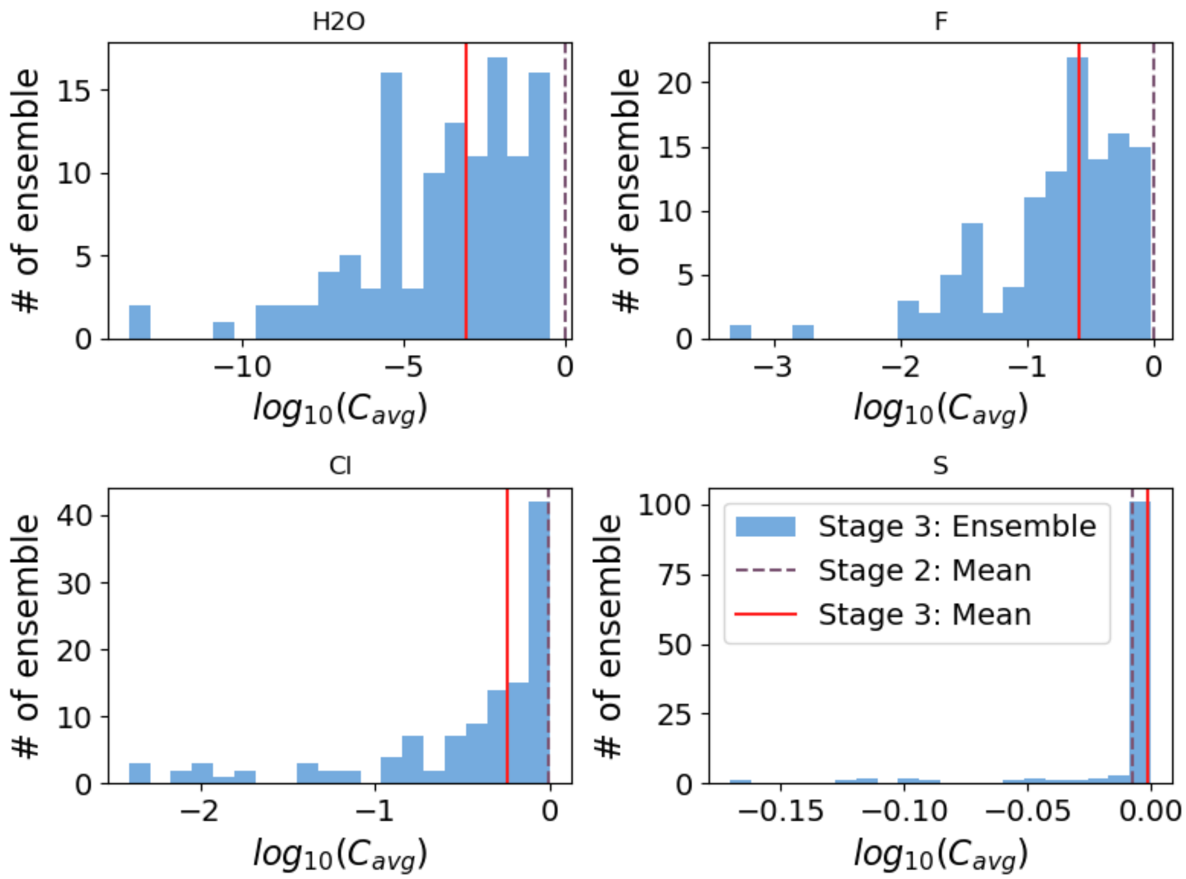

Figure S14. Histograms of the logarithmic average volatile concentrations (ppm) for H_2O, F, Cl, and S in green glass beads. The values are derived from the three-stage degassing model using the parameters from the Ensemble Kalman Filter (EnKF) data assimilation with the orange glass beads. Each panel displays the distribution of ensemble model predictions (blue bars) with the mean parameter value indicated by a red vertical line. The purple dashed lines indicate the mean value of the concentrations at the end of Stage 2 (free flight). Most of the volatile will be lost during the third stage (cooling on lunar surface).

Table S1. The thermal parameters for diffusivities used in the models. Values for D_0 and E_a were obtained by conducting linear regression of diffusion data in anhydrous basalt (Fig. S2) from Zhang et al.[8], Newcombe et al.[7], Alletti et al.[6], and Freda et al.[5].

Component	D_0 (m^2/s)	E_a (kJ/mol)	D at 1330 °C (m^2/s)
H_2O	3.51×10^{-3}	209.5	5.26×10^{-10}
F	7.75×10^{-5}	190.9	4.65×10^{-11}
Cl	1.03×10^{-3}	228.8	3.59×10^{-11}
S	4.93×10^{-4}	237.4	9.05×10^{-12}

Table S2. The parameters of the Ensemble Kalman Filter data assimilation for the three-stage degassing model of the glass beads and the melt embayment.

Parameter	Symbol	Unit	Glass beads		Melt Embayment	
			Initial Guess	Best-fit	Initial Guess	Best-fit
Cooling time scale (Stage 2: free flight)	τ_c	s	$10^{2\sim3}$	$10^{2.56\pm1.15}$	$10^{2\sim3}$	$10^{2.60\pm0.56}$
Glass bead radius	a	μm	50~200	119 ± 78	N/A	N/A
Ratio between diffusion and evaporation timescales (L)						
H_2O	L_{H_2O}	1	$10^{-7\sim-5}$	$10^{-6.44\pm0.59}$	$10^{-6\sim-4}$	$10^{-5.72\pm0.02}$
F	L_F	1	$10^{-7\sim-5}$	$10^{-6.06\pm0.52}$	$10^{-4\sim-2}$	$10^{-5.27\pm0.04}$
Cl	L_{Cl}	1	$10^{-7\sim-5}$	$10^{-5.42\pm0.67}$	$10^{-4\sim-2}$	$10^{-2.24\pm1.04}$
S	L_S	1	$10^{-7\sim-5}$	$10^{-6.45\pm1.02}$	$10^{-6\sim-4}$	$10^{-4.69\pm0.10}$
Cooling time scale (Stage 3: lunar surface)	t_2	s	$10^{6\sim10}$	$10^{8.02\pm0.87}$	$10^{6\sim12}$	$10^{8.36\pm0.01}$
Initial S concentration	S_0	ppm	100~400	$10^{2.56\pm0.19}$	N/A	N/A

* The best-fit parameter distributions (mean values with the 95% confidence interval) are calculated using EnKF data assimilation.
